# {4-Hy­droxy-*N*′-[(2*E*,3*Z*)-4-oxido-4-phenyl­but-3-en-2-yl­idene]benzo­hydrazidato}dimethyl­tin(IV)

**DOI:** 10.1107/S1600536811023506

**Published:** 2011-06-22

**Authors:** Md. Abu Affan, Norrihan B. Sam, Fasihuddin B. Ahmad, Fraser White, Edward R. T. Tiekink

**Affiliations:** a Faculty of Resource Science and Technology, Universiti Malaysia Sarawak, 94300 Kota Samarahan, Sarawak, Malaysia; b Agilent Technologies UK Ltd, 10 Mead Road, Oxford Industrial Park, Oxford OX5 1QU, England; c Department of Chemistry, University of Malaya, 50603 Kuala Lumpur, Malaysia

## Abstract

The Sn^IV^ atom in the title compound, [Sn(CH_3_)_2_(C_17_H_14_N_2_O_3_)], is five-coordinated within a C_2_N_2_O donor set provided by the *N*,*N*,*O*-tridentate ligand and two methyl groups. The resultant coordination geometry is inter­mediate between trigonal-bipyramidal and square-pyramidal. In the crystal, supra­molecular zigzag chains propagating along the *c*- axis direction are mediated by O—H⋯O hydrogen bonds, and weak C—H⋯π inter­actions consolidate the packing.

## Related literature

For background to the biological inter­est of related compounds, see: Affan *et al.* (2010 ▶). For related structures, see: Affan *et al.* (2009 ▶, 2011 ▶). For additional structural analysis, see: Addison *et al.* (1984 ▶).
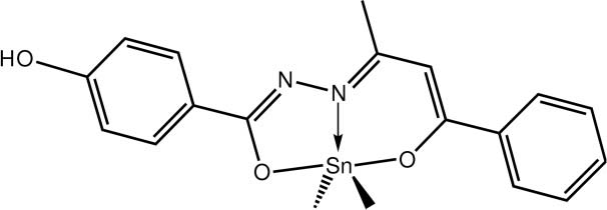


## Experimental

### 

#### Crystal data

[Sn(CH_3_)_2_(C_17_H_14_N_2_O_3_)]
*M* *_r_* = 443.06
Monoclinic, 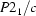

*a* = 8.0784 (2) Å
*b* = 20.5410 (5) Å
*c* = 11.1678 (3) Å
β = 93.025 (2)°
*V* = 1850.58 (8) Å^3^
*Z* = 4
Cu *K*α radiation
μ = 11.15 mm^−1^
*T* = 150 K
0.22 × 0.16 × 0.10 mm

#### Data collection

Agilent SuperNova Dual diffractometer with an Atlas detector
Absorption correction: analytical (*CrysAlis PRO*; Agilent, 2011 ▶) *T*_min_ = 0.696, *T*_max_ = 0.822
5718 measured reflections
3124 independent reflections
2690 reflections with *I* > 2σ(*I*)
*R*_int_ = 0.038

#### Refinement

*R*[*F* ^2^ > 2σ(*F* ^2^)] = 0.032
*wR*(*F* ^2^) = 0.081
*S* = 1.00
3124 reflections
230 parameters
H-atom parameters constrained
Δρ_max_ = 0.76 e Å^−3^
Δρ_min_ = −0.72 e Å^−3^

### 

Data collection: *CrysAlis PRO* (Agilent, 2011 ▶); cell refinement: *CrysAlis PRO*; data reduction: *CrysAlis PRO*; program(s) used to solve structure: *SHELXS97* (Sheldrick, 2008 ▶); program(s) used to refine structure: *SHELXL97* (Sheldrick, 2008 ▶); molecular graphics: *ORTEP-3* (Farrugia, 1997 ▶) and *DIAMOND* (Brandenburg, 2006 ▶); software used to prepare material for publication: *PLATON* (Spek, 2009 ▶) and *publCIF* (Westrip, 2010 ▶).

## Supplementary Material

Crystal structure: contains datablock(s) global, I. DOI: 10.1107/S1600536811023506/hb5915sup1.cif

Structure factors: contains datablock(s) I. DOI: 10.1107/S1600536811023506/hb5915Isup2.hkl

Additional supplementary materials:  crystallographic information; 3D view; checkCIF report

## Figures and Tables

**Table:** 

Sn—O1	2.156 (3)
Sn—O3	2.099 (3)
Sn—N2	2.148 (3)
Sn—C18	2.105 (4)
Sn—C19	2.112 (4)

**Table:** 

O1—Sn—O3	155.08 (10)
C18—Sn—C19	124.65 (18)

**Table 2:** Hydrogen-bond geometry (Å, °) *Cg*1, *Cg*2 and *Cg*3 are the centroids of the C12–C17, Sn,O1,C1,N1,N2 and C2–C7 rings, respectively.

*D*—H⋯*A*	*D*—H	H⋯*A*	*D*⋯*A*	*D*—H⋯*A*
O2—H2*o*⋯O1^i^	0.84	1.91	2.702 (3)	156
C4—H4⋯*Cg*1^ii^	0.95	2.91	3.624 (4)	133
C9—H9c⋯*Cg*2^iii^	0.98	2.88	3.777 (4)	152
C16—H16⋯*Cg*3^iv^	0.95	2.89	3.668 (4)	140

Symmetry codes: (i) 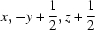; (ii) 
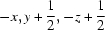
; (iii) 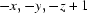; (iv) 
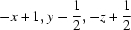
.

**Fig. 1.**
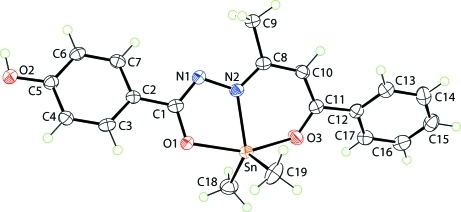
The molecular structure of (I) showing displacement ellipsoids at the 50% probability level.

**Fig. 2.**
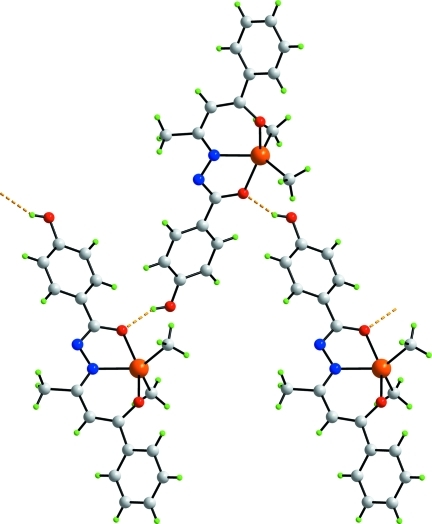
A view of the supramolecular chain aligned along [001] in (I). The O—H···O hydrogen bonds are shown as orange dashed lines.

**Fig. 3.**
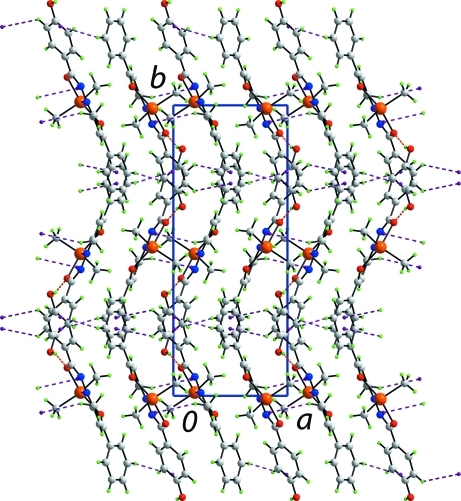
A view in projection down the c axis of the crystal packing in (I). The O—H···O hydrogen bonds and C—H···π interactions are shown as orange and purple dashed lines, respectively.

## supplementary crystallographic information

### Comment

The title compound, (I), was examined in connection with on-going structural studies (Affan *et al.*, 2010) of organotin derivatives of biological interest (Affan *et al.*, 2009), and compliments the structure of the diphenyltin analogue (Affan *et al.*, 2011).

The Sn atom in (I), Fig. 1, is five-coordinated by the tridentate ligand and two methyl groups, Table 1. The resulting C_2_NO_2_ donor set defines a coordination geometry intermediate between square pyramidal and trigonal bipyramidal geometry. This is quantified by the value of τ = 0.51 which compare to the τ values of 0.0 and 1.0 for ideal square pyramidal and trigonal bipyramidal geometries, respectively (Addison *et al.*, 1984). For comparison, the values of τ for the two independent molecules in the structure of the diphenyltin analogue are 0.55 and 0.47 (Affan *et al.*, 2011).

While the five-membered SnCN_2_O chelate ring is almost planar with a r.m.s. deviation = 0.063 Å [max. deviations of 0.039 (1) and -0.052 (2) Å for the Sn and O1 atoms, respectively], there is considerable distortion in the SnC_3_NO six-membered chelate [r.m.s. deviation = 0.226 Å] with the Sn and O3 atoms lying -0.209 (1) and 0.245 (3) Å out of the least-squares plane. Each of the benzene rings is twisted out of the plane from the adjacent chelate ring as seen in the O1—C1—C2—C3 and O3—C11—C12—C13 torsion angles of 13.7 (5) and -150.4 (4)°, respectively. The dihedral angle between the two benzene rings is 68.14 (18) °, indicating a twist in the tridentate ligand.

The crystal packing is dominated by O—H···O hydrogen bonding, Table 2, which leads to a zigzag supramolecular chain along the *c* axis, Fig. 2. These are consolidated in the crystal structure by C—H···π interactions, Table 2.

### Experimental

Benzoylacetone 4-hydroxybenzhydrazone (0.59 g, 2 mmol) was dissolved in distilled methanol (20 ml) under a nitrogen atmosphere. Potassium hydroxide (0.23 g, 4 mmol) dissolved in methanol (10 ml) was added drop wise to the solution. The colour of the solution changed from yellow to orange. The resulting mixture was refluxed for 1 h and then treated with dimethyltin dichloride (0.439 g, 2 mmol) in distilled methanol (10 ml). The resulting mixture was heated under reflux conditions for 4 h and allowed to cool to room temperature. Potassium chloride (KCl) was removed *via* filtration. The filtrate was evaporated to dryness using a rotary evaporator to yield yellow microcrystals. The microcrystals were filtered off and washed with ethanol and dried *in vacuo* over P_2_O_5_ overnight. Yellow blocks of (I) were obtained by slow evaporation of its acetone solution at room temperature. Yield: 0.94 g, 75%. *M*.pt.: 504–506 K. IR (ν_max_, cm^-1^, KBr): 3569 (OH), 1591 (C═N—N═C), 949 (N—N), 562 (Sn—C), 525 (Sn—O), 447 (Sn—N).

### Refinement

Carbon-bound H-atoms were placed in calculated positions (O—H = 0.84 Å; C—H = 0.95 to 0.98 Å) and were included in the refinement in the riding model approximation with *U*_iso_(H) set to 1.2-*U*_eq_(C) and 1.5-*U*_eq_(O, methyl-C).

### Crystal data

**Table:**

[Sn(CH_3_)_2_(C_17_H_14_N_2_O_3_)]	*F*(000) = 888
*M**_r_* = 443.06	*D*_x_ = 1.590 Mg m^−^^3^
Monoclinic, *P*2_1_/*c*	Cu *K*α radiation, λ = 1.54184 Å
Hall symbol: -P 2ybc	Cell parameters from 3495 reflections
*a* = 8.0784 (2) Å	θ = 4.0–74.2°
*b* = 20.5410 (5) Å	µ = 11.15 mm^−^^1^
*c* = 11.1678 (3) Å	*T* = 150 K
β = 93.025 (2)°	Block, yellow
*V* = 1850.58 (8) Å^3^	0.22 × 0.16 × 0.10 mm
*Z* = 4	

### Data collection

**Table:**

Agilent SuperNova Dual diffractometer with an Atlas detector	3124 independent reflections
Radiation source: SuperNova (Cu) X-ray Source	2690 reflections with *I* > 2σ(*I*)
Mirror	*R*_int_ = 0.038
ω scans	θ_max_ = 65.0°, θ_min_ = 4.5°
Absorption correction: analytical (*CrysAlis PRO*; Agilent, 2011)	*h* = −6→9
*T*_min_ = 0.696, *T*_max_ = 0.822	*k* = −24→24
5718 measured reflections	*l* = −13→13

### Refinement

**Table:**

Refinement on *F*^2^	Primary atom site location: structure-invariant direct methods
Least-squares matrix: full	Secondary atom site location: difference Fourier map
*R*[*F*^2^ > 2σ(*F*^2^)] = 0.032	Hydrogen site location: inferred from neighbouring sites
*wR*(*F*^2^) = 0.081	H-atom parameters constrained
*S* = 1.00	*w* = 1/[σ^2^(*F*_o_^2^) + (0.0384*P*)^2^] where *P* = (*F*_o_^2^ + 2*F*_c_^2^)/3
3124 reflections	(Δ/σ)_max_ < 0.001
230 parameters	Δρ_max_ = 0.76 e Å^−^^3^
0 restraints	Δρ_min_ = −0.72 e Å^−^^3^

### Special details

**Table:**

Experimental. Agilent Technologies (2011) *CrysAlis PRO* Software system, version 1.171.34.49, Agilent Technologies UK Ltd, Oxford, UK
Geometry. All s.u.'s (except the s.u. in the dihedral angle between two l.s. planes) are estimated using the full covariance matrix. The cell s.u.'s are taken into account individually in the estimation of s.u.'s in distances, angles and torsion angles; correlations between s.u.'s in cell parameters are only used when they are defined by crystal symmetry. An approximate (isotropic) treatment of cell s.u.'s is used for estimating s.u.'s involving l.s. planes.
Refinement. Refinement of *F*^2^ against ALL reflections. The weighted *R*-factor *wR* and goodness of fit *S* are based on *F*^2^, conventional *R*-factors *R* are based on *F*, with *F* set to zero for negative *F*^2^. The threshold expression of *F*^2^ > 2σ(*F*^2^) is used only for calculating *R*-factors(gt) *etc*. and is not relevant to the choice of reflections for refinement. *R*-factors based on *F*^2^ are statistically about twice as large as those based on *F*, and *R*- factors based on ALL data will be even larger.

### Fractional atomic coordinates and isotropic or equivalent isotropic displacement parameters (Å^2^)

**Table:**

	*x*	*y*	*z*	*U*_iso_*/*U*_eq_	
Sn	0.18670 (3)	0.011355 (13)	0.22279 (2)	0.02375 (11)	
O1	0.0690 (3)	0.09638 (13)	0.2930 (2)	0.0267 (6)	
O2	−0.0780 (3)	0.34947 (13)	0.5942 (2)	0.0309 (6)	
H2o	−0.0354	0.3553	0.6636	0.046*	
O3	0.3618 (3)	−0.06412 (14)	0.2237 (2)	0.0333 (7)	
N1	0.1880 (4)	0.06513 (16)	0.4764 (3)	0.0242 (7)	
N2	0.2394 (4)	0.01068 (16)	0.4134 (3)	0.0232 (7)	
C1	0.1054 (4)	0.10630 (19)	0.4079 (3)	0.0230 (8)	
C2	0.0519 (4)	0.16830 (19)	0.4610 (3)	0.0208 (8)	
C3	−0.0628 (5)	0.2084 (2)	0.3999 (3)	0.0263 (9)	
H3	−0.1131	0.1942	0.3256	0.032*	
C4	−0.1051 (5)	0.2685 (2)	0.4452 (3)	0.0275 (9)	
H4	−0.1835	0.2952	0.4020	0.033*	
C5	−0.0331 (5)	0.28957 (19)	0.5535 (3)	0.0240 (8)	
C6	0.0800 (4)	0.2492 (2)	0.6178 (3)	0.0250 (8)	
H6	0.1282	0.2631	0.6929	0.030*	
C7	0.1213 (4)	0.18929 (19)	0.5719 (3)	0.0245 (8)	
H7	0.1975	0.1621	0.6159	0.029*	
C8	0.2979 (5)	−0.0386 (2)	0.4775 (3)	0.0247 (8)	
C9	0.3027 (5)	−0.0339 (2)	0.6122 (3)	0.0311 (9)	
H9A	0.3736	0.0027	0.6385	0.047*	
H9B	0.3474	−0.0744	0.6472	0.047*	
H9C	0.1903	−0.0269	0.6385	0.047*	
C10	0.3544 (5)	−0.0971 (2)	0.4265 (3)	0.0268 (9)	
H10	0.3750	−0.1326	0.4797	0.032*	
C11	0.3827 (5)	−0.1079 (2)	0.3075 (3)	0.0255 (9)	
C12	0.4463 (4)	−0.1711 (2)	0.2669 (3)	0.0243 (8)	
C13	0.4076 (5)	−0.2292 (2)	0.3228 (4)	0.0286 (9)	
H13	0.3440	−0.2285	0.3919	0.034*	
C14	0.4607 (5)	−0.2880 (2)	0.2787 (4)	0.0340 (10)	
H14	0.4317	−0.3274	0.3169	0.041*	
C15	0.5565 (5)	−0.2900 (2)	0.1787 (4)	0.0365 (10)	
H15	0.5938	−0.3304	0.1489	0.044*	
C16	0.5966 (5)	−0.2323 (2)	0.1232 (4)	0.0351 (10)	
H16	0.6629	−0.2332	0.0554	0.042*	
C17	0.5410 (5)	−0.1731 (2)	0.1658 (4)	0.0295 (9)	
H17	0.5674	−0.1339	0.1260	0.035*	
C18	0.3273 (6)	0.0647 (2)	0.1034 (4)	0.0379 (11)	
H18A	0.4406	0.0701	0.1380	0.057*	
H18B	0.2767	0.1076	0.0896	0.057*	
H18C	0.3302	0.0413	0.0271	0.057*	
C19	−0.0261 (6)	−0.0439 (2)	0.1720 (4)	0.0451 (12)	
H19A	−0.0003	−0.0739	0.1074	0.068*	
H19B	−0.1158	−0.0146	0.1439	0.068*	
H19C	−0.0611	−0.0688	0.2411	0.068*	

### Atomic displacement parameters (Å^2^)

**Table:**

	*U*^11^	*U*^22^	*U*^33^	*U*^12^	*U*^13^	*U*^23^
Sn	0.02822 (16)	0.02438 (17)	0.01860 (15)	0.00018 (11)	0.00060 (10)	−0.00033 (10)
O1	0.0341 (15)	0.0238 (15)	0.0217 (14)	0.0056 (12)	−0.0027 (10)	−0.0038 (11)
O2	0.0410 (17)	0.0274 (16)	0.0237 (15)	0.0055 (13)	−0.0045 (12)	−0.0050 (12)
O3	0.0419 (17)	0.0311 (16)	0.0277 (15)	0.0123 (14)	0.0084 (12)	0.0059 (13)
N1	0.0271 (17)	0.0232 (18)	0.0222 (16)	0.0013 (14)	0.0008 (13)	−0.0027 (14)
N2	0.0250 (16)	0.0260 (18)	0.0192 (16)	0.0003 (14)	0.0042 (13)	0.0003 (14)
C1	0.0199 (18)	0.029 (2)	0.0200 (19)	−0.0050 (16)	0.0023 (14)	0.0008 (17)
C2	0.0194 (18)	0.024 (2)	0.0195 (18)	−0.0024 (16)	0.0012 (14)	0.0012 (15)
C3	0.025 (2)	0.033 (2)	0.0200 (19)	−0.0033 (18)	−0.0027 (15)	−0.0012 (17)
C4	0.029 (2)	0.029 (2)	0.0241 (19)	0.0065 (18)	−0.0018 (15)	−0.0001 (17)
C5	0.026 (2)	0.025 (2)	0.0218 (19)	−0.0037 (17)	0.0009 (14)	−0.0006 (16)
C6	0.025 (2)	0.029 (2)	0.0204 (18)	−0.0059 (17)	−0.0005 (15)	−0.0040 (17)
C7	0.0220 (19)	0.028 (2)	0.0238 (19)	−0.0009 (17)	−0.0001 (14)	0.0034 (17)
C8	0.0236 (19)	0.029 (2)	0.0211 (19)	−0.0008 (18)	0.0019 (14)	0.0010 (17)
C9	0.044 (2)	0.029 (2)	0.020 (2)	0.002 (2)	0.0029 (17)	−0.0001 (18)
C10	0.030 (2)	0.027 (2)	0.0237 (19)	0.0031 (18)	0.0024 (15)	0.0033 (17)
C11	0.0230 (19)	0.027 (2)	0.026 (2)	−0.0023 (17)	0.0008 (15)	−0.0008 (17)
C12	0.0177 (18)	0.028 (2)	0.027 (2)	0.0000 (16)	−0.0033 (14)	−0.0035 (17)
C13	0.023 (2)	0.032 (2)	0.031 (2)	−0.0025 (18)	0.0005 (16)	−0.0011 (18)
C14	0.027 (2)	0.027 (2)	0.047 (3)	−0.0004 (19)	−0.0059 (18)	−0.001 (2)
C15	0.031 (2)	0.036 (3)	0.041 (3)	0.009 (2)	−0.0062 (18)	−0.011 (2)
C16	0.028 (2)	0.046 (3)	0.031 (2)	0.008 (2)	0.0005 (17)	−0.004 (2)
C17	0.026 (2)	0.034 (2)	0.028 (2)	0.0020 (19)	0.0032 (15)	0.0007 (19)
C18	0.043 (3)	0.040 (3)	0.031 (2)	−0.008 (2)	0.0029 (18)	0.003 (2)
C19	0.045 (3)	0.042 (3)	0.048 (3)	−0.011 (2)	0.006 (2)	−0.020 (2)

### Geometric parameters (Å, °)

**Table:**

Sn—O1	2.156 (3)	C8—C9	1.506 (5)
Sn—O3	2.099 (3)	C9—H9A	0.9800
Sn—N2	2.148 (3)	C9—H9B	0.9800
Sn—C18	2.105 (4)	C9—H9C	0.9800
Sn—C19	2.112 (4)	C10—C11	1.377 (5)
O1—C1	1.317 (4)	C10—H10	0.9500
O2—C5	1.367 (5)	C11—C12	1.476 (5)
O2—H2O	0.8400	C12—C13	1.390 (6)
O3—C11	1.303 (5)	C12—C17	1.398 (5)
N1—C1	1.300 (5)	C13—C14	1.380 (6)
N1—N2	1.396 (4)	C13—H13	0.9500
N2—C8	1.313 (5)	C14—C15	1.393 (6)
C1—C2	1.479 (5)	C14—H14	0.9500
C2—C3	1.391 (5)	C15—C16	1.384 (6)
C2—C7	1.400 (5)	C15—H15	0.9500
C3—C4	1.384 (6)	C16—C17	1.388 (6)
C3—H3	0.9500	C16—H16	0.9500
C4—C5	1.383 (5)	C17—H17	0.9500
C4—H4	0.9500	C18—H18A	0.9800
C5—C6	1.404 (5)	C18—H18B	0.9800
C6—C7	1.381 (5)	C18—H18C	0.9800
C6—H6	0.9500	C19—H19A	0.9800
C7—H7	0.9500	C19—H19B	0.9800
C8—C10	1.417 (5)	C19—H19C	0.9800
			
O3—Sn—C18	90.09 (15)	C8—C9—H9B	109.5
O3—Sn—C19	98.26 (16)	H9A—C9—H9B	109.5
O1—Sn—O3	155.08 (10)	C8—C9—H9C	109.5
C18—Sn—C19	124.65 (18)	H9A—C9—H9C	109.5
O3—Sn—N2	83.82 (11)	H9B—C9—H9C	109.5
C18—Sn—N2	123.05 (15)	C11—C10—C8	126.7 (4)
C19—Sn—N2	112.24 (16)	C11—C10—H10	116.7
C18—Sn—O1	94.10 (15)	C8—C10—H10	116.7
C19—Sn—O1	99.46 (15)	O3—C11—C10	124.1 (4)
N2—Sn—O1	73.31 (11)	O3—C11—C12	114.8 (3)
C1—O1—Sn	113.6 (2)	C10—C11—C12	121.0 (4)
C5—O2—H2O	109.5	C13—C12—C17	118.8 (4)
C11—O3—Sn	125.1 (2)	C13—C12—C11	121.8 (3)
C1—N1—N2	112.4 (3)	C17—C12—C11	119.3 (4)
C8—N2—N1	116.8 (3)	C14—C13—C12	120.7 (4)
C8—N2—Sn	126.3 (3)	C14—C13—H13	119.7
N1—N2—Sn	116.5 (2)	C12—C13—H13	119.7
N1—C1—O1	123.6 (4)	C13—C14—C15	120.5 (4)
N1—C1—C2	118.4 (3)	C13—C14—H14	119.7
O1—C1—C2	118.0 (3)	C15—C14—H14	119.7
C3—C2—C7	118.5 (4)	C16—C15—C14	119.1 (4)
C3—C2—C1	121.0 (3)	C16—C15—H15	120.4
C7—C2—C1	120.5 (3)	C14—C15—H15	120.4
C4—C3—C2	121.3 (4)	C15—C16—C17	120.6 (4)
C4—C3—H3	119.3	C15—C16—H16	119.7
C2—C3—H3	119.3	C17—C16—H16	119.7
C5—C4—C3	119.9 (4)	C16—C17—C12	120.3 (4)
C5—C4—H4	120.1	C16—C17—H17	119.9
C3—C4—H4	120.1	C12—C17—H17	119.9
O2—C5—C4	117.8 (3)	Sn—C18—H18A	109.5
O2—C5—C6	122.6 (3)	Sn—C18—H18B	109.5
C4—C5—C6	119.6 (4)	H18A—C18—H18B	109.5
C7—C6—C5	120.0 (3)	Sn—C18—H18C	109.5
C7—C6—H6	120.0	H18A—C18—H18C	109.5
C5—C6—H6	120.0	H18B—C18—H18C	109.5
C6—C7—C2	120.7 (4)	Sn—C19—H19A	109.5
C6—C7—H7	119.7	Sn—C19—H19B	109.5
C2—C7—H7	119.7	H19A—C19—H19B	109.5
N2—C8—C10	123.3 (3)	Sn—C19—H19C	109.5
N2—C8—C9	119.0 (4)	H19A—C19—H19C	109.5
C10—C8—C9	117.7 (4)	H19B—C19—H19C	109.5
C8—C9—H9A	109.5		
			
O3—Sn—O1—C1	−17.4 (4)	C3—C4—C5—O2	179.6 (3)
C18—Sn—O1—C1	−116.5 (3)	C3—C4—C5—C6	−1.3 (6)
C19—Sn—O1—C1	117.4 (3)	O2—C5—C6—C7	−179.7 (3)
N2—Sn—O1—C1	6.8 (2)	C4—C5—C6—C7	1.2 (6)
C18—Sn—O3—C11	158.3 (3)	C5—C6—C7—C2	0.3 (6)
C19—Sn—O3—C11	−76.6 (3)	C3—C2—C7—C6	−1.7 (5)
N2—Sn—O3—C11	35.0 (3)	C1—C2—C7—C6	175.6 (3)
O1—Sn—O3—C11	58.3 (4)	N1—N2—C8—C10	179.9 (3)
C1—N1—N2—C8	−168.5 (3)	Sn—N2—C8—C10	8.0 (5)
C1—N1—N2—Sn	4.2 (4)	N1—N2—C8—C9	1.2 (5)
O3—Sn—N2—C8	−24.1 (3)	Sn—N2—C8—C9	−170.7 (3)
C18—Sn—N2—C8	−110.2 (3)	N2—C8—C10—C11	11.3 (6)
C19—Sn—N2—C8	72.3 (3)	C9—C8—C10—C11	−170.0 (4)
O1—Sn—N2—C8	165.9 (3)	Sn—O3—C11—C10	−30.7 (5)
O3—Sn—N2—N1	164.0 (3)	Sn—O3—C11—C12	151.4 (3)
C18—Sn—N2—N1	78.0 (3)	C8—C10—C11—O3	0.6 (6)
C19—Sn—N2—N1	−99.5 (3)	C8—C10—C11—C12	178.3 (4)
O1—Sn—N2—N1	−5.9 (2)	O3—C11—C12—C13	−150.4 (4)
N2—N1—C1—O1	2.4 (5)	C10—C11—C12—C13	31.7 (5)
N2—N1—C1—C2	−176.3 (3)	O3—C11—C12—C17	26.3 (5)
Sn—O1—C1—N1	−7.7 (5)	C10—C11—C12—C17	−151.6 (4)
Sn—O1—C1—C2	171.1 (2)	C17—C12—C13—C14	−0.5 (6)
N1—C1—C2—C3	−167.5 (3)	C11—C12—C13—C14	176.2 (4)
O1—C1—C2—C3	13.7 (5)	C12—C13—C14—C15	1.1 (6)
N1—C1—C2—C7	15.3 (5)	C13—C14—C15—C16	−0.6 (6)
O1—C1—C2—C7	−163.5 (3)	C14—C15—C16—C17	−0.6 (6)
C7—C2—C3—C4	1.7 (6)	C15—C16—C17—C12	1.3 (6)
C1—C2—C3—C4	−175.6 (3)	C13—C12—C17—C16	−0.7 (6)
C2—C3—C4—C5	−0.2 (6)	C11—C12—C17—C16	−177.5 (4)

### Hydrogen-bond geometry (Å, °)

**Table:**

Cg1, Cg2 and Cg3 are the centroids of the C12–C17, Sn,O1,C1,N1,N2 and C2–C7 rings, respectively.

**Table:**

*D*—H···*A*	*D*—H	H···*A*	*D*···*A*	*D*—H···*A*
O2—H2o···O1^i^	0.84	1.91	2.702 (3)	156
C4—H4···Cg1^ii^	0.95	2.91	3.624 (4)	133
C9—H9c···Cg2^iii^	0.98	2.88	3.777 (4)	152
C16—H16···Cg3^iv^	0.95	2.89	3.668 (4)	140

Symmetry codes: (i) *x*, −*y*+1/2, *z*+1/2; (ii) −*x*, *y*+1/2, −*z*+1/2; (iii) −*x*, −*y*, −*z*+1; (iv) −*x*+1, *y*−1/2, −*z*+1/2.
